# Peak Plasma Levels of mtDNA Serve as a Predictive Biomarker for COVID-19 in-Hospital Mortality

**DOI:** 10.3390/jcm11237161

**Published:** 2022-12-01

**Authors:** Fabian Edinger, Sophia Edinger, Christian Koch, Melanie Markmann, Matthias Hecker, Michael Sander, Emmanuel Schneck

**Affiliations:** 1 Department of Anaesthesiology, Critical Care Medicine and Pain Therapy, University Hospital of Giessen, Justus-Liebig-University, 35392 Giessen, Germany; 2 Department of Internal Medicine, Pulmonary and Critical Care Medicine, University Hospital of Giessen, Klinikstr. 33, 35392 Giessen, Germany

**Keywords:** SARS-CoV2, immunothrombosis, intensive care unit, nucleic acids, biomarker

## Abstract

Several predictive biomarkers for coronavirus disease (COVID-19)-associated mortality in critically ill patients have been described. Although mitochondrial DNA (mtDNA) is elevated in patients with COVID-19, the association with coagulation function and its predictive power for mortality is unclear. Accordingly, this study investigates the predictive power of mtDNA for in-hospital mortality in critically ill patients with COVID-19, and whether combining it with thromboelastographic parameters can increase its predictive performance. This prospective explorative study included 29 patients with COVID-19 and 29 healthy matched controls. mtDNA encoding for NADH dehydrogenase 1 (ND1) was quantified using a quantitative polymerase chain reaction analysis, while coagulation function was evaluated using thromboelastometry and impedance aggregometry. Receiver operating characteristic (ROC) curves were used for the prediction of in-hospital mortality. Within the first 24 h, the plasma levels of mtDNA peaked significantly (controls: 65 (28–119) copies/µL; patients: 281 (110–805) at t_0_, 403 (168–1937) at t_24_, and 467 (188–952) copies/µL at t_72_; controls vs. patients: *p* = 0.02 at t_0_, *p* = 0.03 at t_24_, and *p* = 0.44 at t_72_). The mtDNA levels at t_24_ showed an excellent predictive performance for in-hospital mortality (area under the ROC curve: 0.90 (0.75–0.90)), which could not be improved by the combination with thromboelastometric or aggregometric parameters. Critically ill patients with COVID-19 present an early increase in the plasma levels of ND1 mtDNA, lasting over 24 h. They also show impairments in platelet function and fibrinolysis, as well as hypercoagulability, but these do not correlate with the plasma levels of fibrinogen. The peak plasma levels of mtDNA can be used as a predictive biomarker for in-hospital mortality; however, the combination with coagulation parameters does not improve the predictive validity.

## 1. Introduction

The coronavirus disease (COVID-19) pandemic has become a global health crisis. To date, more than 598 million people have been infected with severe acute respiratory syndrome coronavirus 2 (SARS-CoV-2), and almost 6.4 million individuals have died worldwide [1]. COVID-19 has been recognized as a systemic inflammatory disease affecting multiple organ systems, such as the lungs, heart, kidneys, liver, and brain [2]. In particular, COVID-19-induced endothelial dysfunction contributes to vascular inflammation, which is closely connected to immunothrombosis activation [3,4].

Apart from activating the endothelium in association with cytokine activity, SARS-CoV-2 also directly invades the endothelial cells after binding to angiotensin-converting enzyme 2, resulting in diffuse vascular inflammation [5,6,7]. Briefly, the prothrombotic molecules thrombin, plasminogen activator inhibitor-1, von Willebrand factor, tissue factor and platelet-activating factor are released from activated endothelial cells, which finally results in COVID-19-associated coagulopathy [3].

Many efforts have been made to identify predictive biomarkers for severe COVID-19, such as the patient’s lymphocyte count or neurofilament light chain levels [8,9,10]. In this context, mitochondrial DNA (mtDNA) serves as a reasonable focal point, as it is linked to immunothrombosis during systemic inflammatory reactions, such as severe trauma, major surgery-related inflammation and sepsis. It has already been reported to be related to non-COVID-19-associated intensive care unit (ICU) mortality [11,12,13,14]. Recently, a study showed that elevated mtDNA levels might be associated with poor outcomes in patients with COVID-19 [15]. However, this study included all patients hospitalized with COVID-19, rather than focusing specifically on those with critical illness needing ICU treatment.

Thromboelastographic parameters are of increasing interest for predicting thrombotic events, due to their rapid processing time and wide-spread availability [16,17,18,19,20,21,22,23]. COVID-19-associated coagulopathy is reflected by increased maximum clot firmness (MCF) in extrinsic (extrinsically activated thromboelastometry [EXTEM]) and fibrinogen-dependent (fibrinogen-based thromboelastometry [FIBTEM]) ROTEM assays [16,18,19,22,23]. Furthermore, a higher MCF in the EXTEM has been detected in critically ill patients compared with non-critically ill patients with COVID-19 [24].

Thromboelastometry has been investigated as a tool for predicting coagulopathy, but not for mortality [15,25]. To date, the role of platelet dysfunction in the prediction of mortality has not yet been investigated. Therefore, this explorative study evaluates the predictive power of mtDNA for COVID-19-associated mortality in a cohort of critically ill patients, and whether the combination of thromboelastographic parameters and mtDNA levels can be used as a multi-parametric biomarker for COVID-19-associated mortality.

## 2. Materials and Methods

### 2.1. Study Design

This single-center, prospective, observational proof-of-concept study included 29 patients treated at an ICU with COVID-19 (positive polymerase chain reaction [PCR] test result for SARS-CoV-2 [Roche Diagnostics, Rotkreuz, Switzerland]) and 29 healthy controls, who were not treated at an ICU but matched in age (age difference of up to 5 years), sex and pre-existing disorders. Lacking data on free-circulating mtDNA in patients suffering from COVID-19 to the time point of study planning, a sample size calculation was not feasible; thus, this study is explorative. All patients were enrolled between November 2020 and May 2021 at the University Hospital of Giessen.

The present study was registered at the German Clinical Trials Register (trial code: DRKS00030005), and approved by our local ethics committee (Justus-Liebig-University of Giessen, trial code: 65/20). The study was conducted in accordance with the Declaration of Helsinki and the Strengthening the Reporting of Observational Studies in Epidemiology guidelines. The inclusion criteria consisted of having been admitted to the surgical ICU of the University Hospital of Giessen within 24 h, having a positive SARS-CoV-2 PCR test, being of legal age and giving informed consent, which was obtained through the legal representative when applicable. The exclusion criteria comprised having a negative SARS-CoV-2 PCR test, a positive proof of SARS-CoV-2 older than 24 h, or being under 18 years of age.

### 2.2. Sample Processing

While the blood of patients with COVID-19 was drawn for sampling through an arterial or central line, that of the controls was drawn through the cubital vein. The blood samples were collected in ethylenediaminetetraacetic acid tubes for enzyme-linked immunosorbent assay (ELISA) and PCR analyses. Citrate probes and hirudin tubes were used for thromboelastographic and platelet impedance aggregometric analyses, respectively. The blood samples of the patients with COVID-19 were collected at inclusion (t_0_), after 24 h (t_24_) and after 72 h (t_72_), while those of the controls were collected only once. The thromboelastometric and platelet impedance aggregometric analyses were performed directly. The plasma samples were stored at −80 °C for further analyses. Clinical data were extracted from the local patient data management system (IMESO GmbH, Giessen, Germany).

### 2.3. Coagulation Analysis

While thromboelastography was conducted using the ROTEM Delta analyzer (Tem Innovations GmbH, Munich, Germany), whole blood ristocetin-induced platelet impedance aggregometry was performed using the Multiplate Analyzer (Multiplate, Roche Diagnostics, Mannheim, Germany). Thromboelastography was performed using intrinsically activated thromboelastometry (INTEM) and EXTEM reagents to evaluate the coagulatory function of the intrinsic and extrinsic pathways, respectively, and FIBTEM and aprotinin-based thromboelastometry (APTEM) reagents were performed to investigate fibrinogen-dependent coagulation and the extent of fibrinolysis, respectively. Initially, the citrate samples were treated with the star-TEM reagent to recalcify the blood, and with the above-mentioned reagents. The extrinsic coagulation pathway was activated by the tissue factor by adding the EXTEM reagent, while the intrinsic coagulation pathway was activated by phospholipid and ellagic acid by adding the INTEM reagent. Additionally, fibrinogen-dependent coagulation was stimulated using the cytochalasin D-containing FIBTEM reagent, while fibrinolysis was inhibited using the APTEM reagent. Validated thromboelastographic values for the initiation of coagulation (clotting time in seconds, clot firmness time in seconds, clot strength based on the MCF in millimeters and fibrinolysis based on the maximum lysis (ML) as the percentage of the MCF) were recorded [26].

Impedance aggregometry was used to describe platelet aggregation in the whole blood samples. With the aid of an automatic pipette, either thrombin receptor-activating peptide (TRAP) (TRAPtest, Verum Diagnostica GmbH, Munich, Germany), adenosine diphosphate (ADP) (ADPtest, Verum Diagnostica GmbH) or arachidonic acid (ASPI) (ASPItest, Verum Diagnostica GmbH) was added in accordance with the system’s instructions. To describe the aggregation capacity, we recorded the area under the curve (AUC) of the impedance aggregometric parameters [26]. Platelets were stimulated with ASPI, ADP and TRAP-6.

### 2.4. Laboratory Parameters

The laboratory parameters included the leukocyte, thrombocyte, neutrophilic granulocytes and lymphocyte counts, the glomerular filtration rate, the international normalized ratio and the levels of fibrinogen, D-dimer, C-reactive protein (CRP), procalcitonin, interleukin 6, ferritin, creatinine, urea, glutamic oxaloacetic transaminase, glutamic pyruvic transaminase and lactate dehydrogenase. All parameters were measured during routine clinical tests at the local laboratory of the University Hospital of Giessen.

### 2.5. MtDNA Quantification

NADH dehydrogenase 1 (ND1) mtDNA was quantified using a quantitative polymerase chain reaction (qPCR) analysis, as described previously [13,27]. Initially, blood was centrifuged at 200 units of gravity (× *g*) for 10 min at room temperature to isolate the plasma. Thereafter, 100 µL of plasma was diluted with 100 µL of phosphate-buffered saline, and the mixture was centrifuged again at 5000× *g* for 10 min at 4 °C. The supernatant was frozen at −20 °C. After thawing, the mtDNA was purified using a commercial purification kit following the manufacturer’s instructions (QIAquick PCR Purification Kit, Qiagen, Venlo, The Netherlands). The samples were then diluted at a ratio of 1:20 with nuclease-free, deionized–distilled H_2_O before the qPCR analysis. The StepOnePlus cycler (Thermo Fisher Scientific, Waltham, MA, USA) was used to quantify ND1 mtDNA in all samples with the following primers: ND1 mtDNA FW: 5′-CCA CCT CTA GCC TAG CCG TTT A-3′ and ND1 mtDNA RW: 5′-GGG TCA TGA TGG CAG GAG TAA T-3′ (synthesized by Eurofins, Luxembourg).

Next, the results were converted to the number of copies per microliter, according to the method described by Chiu et al., based on a standard curve generated using a human ND1 mtDNA-containing plasmid (OriGene Technologies, Rockville, MD, USA) [28]. Serial dilutions of the corresponding plasmid copy number (30–300,000 copies per PCR reaction) were used, and the number of plasmid copies was calculated using the NanoDrop 2000 spectrophotometer (Thermo Fisher Scientific).

### 2.6. Statistical Analysis

All data were expressed as medians and interquartile ranges (25th–75th percentiles). An analysis of variance was used followed by a post-hoc Bonferroni test to compare the patient and control groups. Spearman’s correlation coefficients were applied for the correlations between the ND1 mtDNA levels and ROTEM analysis results. Receiver operating characteristic (ROC) curves were used to calculate the predictive power of ND1 mtDNA. These analyses primarily aimed to predict in-hospital mortality based on the AUC ROC. AUC ROC values of 0.51–0.69, 0.7–0.79, 0.8–0.89 and ≥0.9 were considered to be poor, adequate, sufficient and excellent, respectively. These values were presented with their corresponding 95% confidence intervals. A *p*-value of ≤0.05 was considered statistically significant. All statistical analyses were performed using the R statistical software (version 3.6.2, 12 December 2019; www.r-project.org).

## 3. Results

The basic and ICU patient characteristics are presented in Table 1, and the laboratory findings can be found in the Supplementary Table S1. All data were shown as medians with interquartile ranges or percentages. Compared with that of the control group, the CRP levels of the COVID-19 patient group were significantly elevated at all timepoints. Furthermore, the leucocytes of the COVID-19 patients were increased compared to the controls at t_72_. Since some of the patients passed away or were transferred to the ward, the analysis consists of 25 (t_24_) and 24 (t_72_) patients, respectively. Moreover, the clinical data of all deceased COVID-19 patients are presented in the Supplementary Table S2.

### 3.1. ND1 mtDNA Quantification

**Figure 1:**
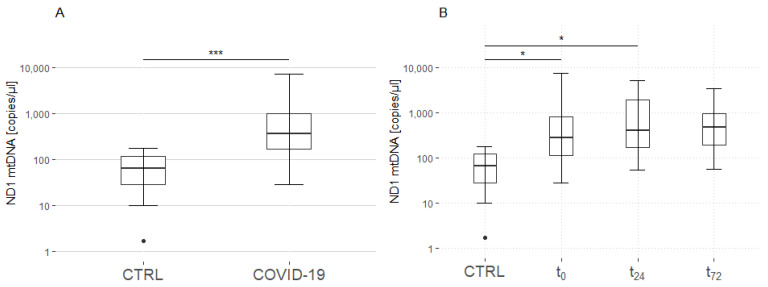
Quantification of ND1 mtDNA. (**A**): Significantly elevated levels of ND1 mtDNA were found in patients with COVID-19 compared with those in matched healthy controls. (**B**): Elevated levels of ND1 mtDNA were found in the COVID-19 patients after admission to the intensive care unit (t_0_) and 24 h thereafter (t_24_), compared with those in the controls. An analysis of the different time points among the COVID-19 patients revealed no significant differences. Asterisks display the degree of statistical significance: *: *p* ≤ 0.05, ***: *p* < 0.001. Abbreviations: COVID-19 = coronavirus disease; CTRL = control group; mtDNA = mitochondrial DNA; ND1 = NADH dehydrogenase 1.

An analysis of the mean of all time points demonstrated that the COVID-19 patient group showed elevated levels of ND1 mtDNA compared with those of the control group (controls: 65 (28–119) copies/µL; patients: 363 (167–987) copies/µL; *p* < 0.001; Figure 1A). An analysis of the single time points indicated that the levels of mtDNA significantly increased during the first 24 h (t_24_) after ICU admission (t_0_). Thereafter, the plasma levels of mtDNA in the patient group continued to increase compared with those of the control group (t_72_), but did not reach statistical significance (controls: 65 (28–119) copies/µL; patients: 281 (110–805) at t_0_, 403 (168–1937) at t_24_ and 467 (188–952) copies/µL at t_72_; controls vs. patients: *p* = 0.02 at t_0_, *p* = 0.03 at t_24_ and *p* = 0.44 at t_72_; Figure 1B). Furthermore, the analysis of the different time points among the COVID-19 patients revealed no significant differences (t_0_ vs. t_24_: *p* = 1.0; t_0_ vs. t_72_: *p* = 1.0; t_24_ vs. t_72_: *p* = 1.0).

A correlation analysis of ND1 mtDNA with thrombocytes and neutrophilic granulocytes revealed only a positive correlation at t_0_ for neutrophilic granulocytes (r = 0.46; *p* = 0.027).

### 3.2. Platelet Impedance Aggregometry

**Figure 2:**
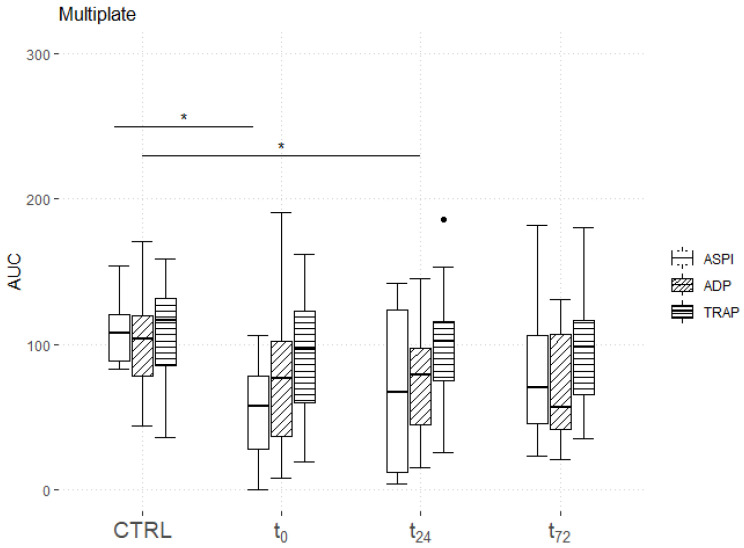
Time course of impedance platelet aggregometry. Patients with COVID-19 presented significantly unsatisfactory AUCs for the ASPI level at t_0_ and the ADP level at t_24_. No significant differences in the TRAP level were found. Asterisks display the degree of statistical significance: *: *p* ≤ 0.05. Abbreviations: ADP = adenosine diphosphate; ASPI = arachidonic acid; AUC = area under the curve; CTRL = control group; TRAP = thrombin receptor-activating peptide.

The expression of ASPI was significantly suppressed in the patient group, compared with the control group, only at t_0_ (controls: 108 (89–121); patients: 58 (29–79) at t_0_, 67 (12–124) at t_24_ and 70 (46–106) at t_72_; *p* = 0.03 at t_0_, *p* = 0.22 at t_24_ and *p* = 0.99 at t_72_; Figure 2). In contrast, the ADP level significantly decreased in the patient group at t_24_ (controls: 104 (79–120); patients: 77 (37–103) at t_0_, 79 (45–98) at t_24_ and 57 (42–107) at t_72_; *p* = 0.13 at t_0_, *p* = 0.04 at t_24_ and *p* = 0.07 at t_72_). The TRAP level did not significantly differ between the two groups, independently of the time points.

### 3.3. Rotational Thromboelastometry

**Figure 3:**
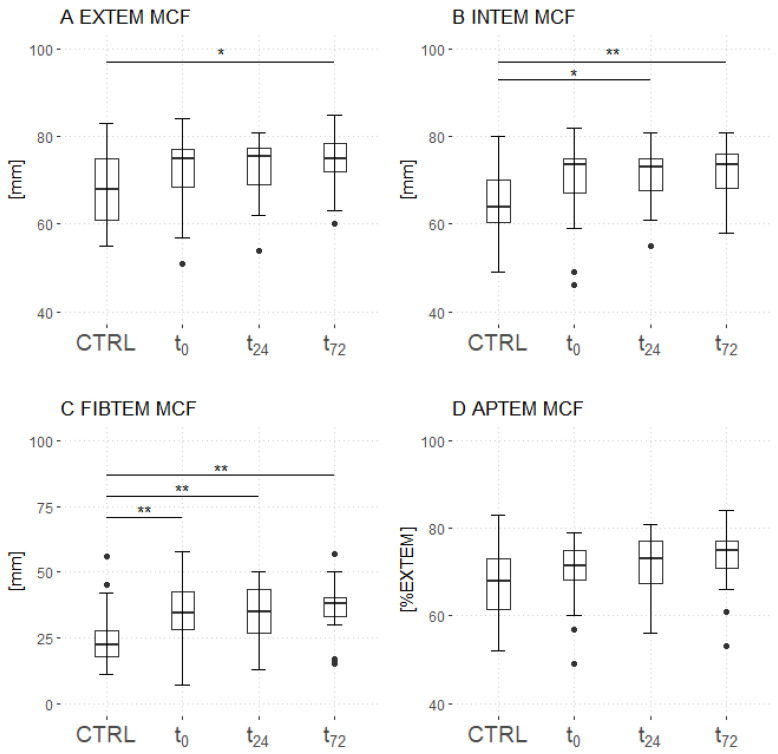
Time course of thromboelastometry. Patients with COVID-19 presented a significantly increased MCF at 72 h (EXTEM (**A**) and INTEM (**B**) assays) and 24 h (INTEM (**B**)) after admission to the ICU compared with the controls. In the FIBTEM assay (**C**), the MCF increased in patients with COVID-19 at all time points compared with that in the controls. No differences were found in the APTEM assay (**D**). Asterisks display the degree of statistical significance: *: *p* ≤ 0.05, **: *p* < 0.01. Abbreviations: APTEM = aprotinin-based thromboelastometry; CTRL = control group; EXTEM = extrinsically activated thromboelastometry; FIBTEM = fibrinogen-based thromboelastometry; INTEM = intrinsically activated thromboelastometry; MCF = maximum clot firmness.

While the MCF was measured increased only at t_72_ (EXTEM and INTEM) and t_24_ (INTEM) (EXTEM: *p* = 0.07 at t_24_ and *p* = 0.01 at t_72_; Figure 3A; INTEM: *p* = 0.04 at t_24_ and *p* = 0.003 at t_72_; Figure 3B), significantly increased values were recorded in the patient group compared with the control group at all timepoints regarding FIBTEM (*p* = 0.009 at t_0_, *p* = 0.005 at t_24_ and *p* = 0.002 at t_72_; Figure 3C). Similar results were measured for the amplitude after 10 (A10) and 20 (A20) minutes regarding INTEM, FIBTEM and APTEM assays (Supplementary Table S3). However, significant differences were measured between COVID-19 and control group in the EXTEM assays at t_24_ (A10) and t_72_ (A10 and A20). These results are pictured in Supplemental Figures S1 and S2.

In the EXTEM, INTEM and FIBTEM assays, the patient group showed significantly decreased ML compared to the control group (all *p* < 0.001). No differences between the groups were captured in the APTEM assay regarding MCF and ML. The ROTEM measurements are detailed in Supplementary Table S3.

Independently of the used thromboelastographic assay and time point, none of the parameters were significantly associated with the ND1 mtDNA level.

### 3.4. Mortality Prediction

**Figure 4:**
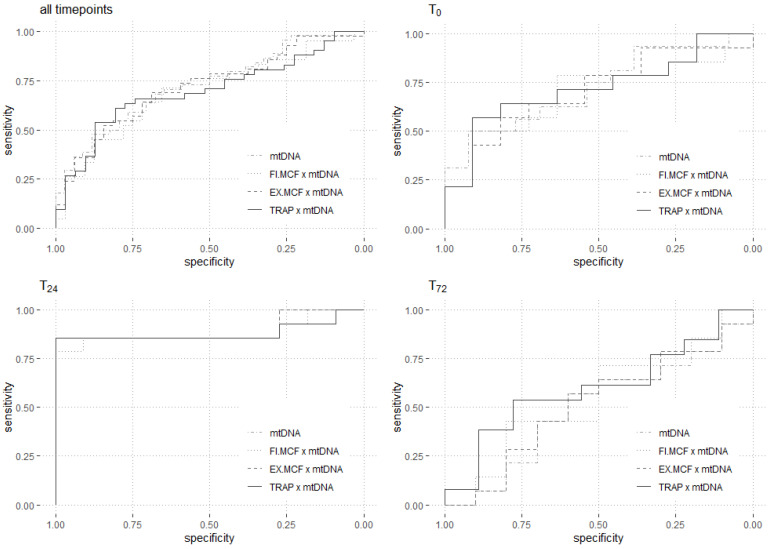
Prediction of in-hospital mortality. The receiver operating characteristic curves for the mtDNA level, FIBTEM MCF × mtDNA level, EXTEM MCF × mtDNA level and TRAP × mtDNA level are shown. The curves are presented for each time point (t_0_, t_24_ and t_72_) individually, as well as for all time points. Abbreviations: EX.MCF = maximum clot firmness in the extrinsically activated thromboelastometry assay; FI.MCF = maximum clot firmness in the fibrinogen-based thromboelastometry assay; mtDNA = mitochondrial DNA; TRAP = thrombin receptor-activating peptide.

The analysis of all time points (t_0_, t_24_ and t_72_) of the patients suffering from COVID-19 demonstrated that the plasma levels of mtDNA showed an adequate predictive validity for in-hospital mortality (AUC ROC = 0.73 (0.61–0.73); Figure 4). Moreover, the analysis at the single time points revealed an excellent prediction of mortality at t_24_ (AUC ROC = 0.90 (0.75–0.90)). The data of the COVID-19 patients at all time points (t_0_, t_24_ and t_72_) of the patients suffering from COVID-19 are detailed in Table 2 and Figure 4. A cut-off threshold for ND1 mtDNA of 420 copies/µL resulted in a sensitivity of 0.86 and a specificity of 1.00.

Based on their significant elevation in the patient group, the analysis focused on FIBTEM MCF and EXTEM MCF. However, these were unable to predict in-hospital mortality (Table 2).

Meanwhile, the platelet impedance aggregometric parameters showed only a poor predictive power for in-hospital mortality. Only the values at t_72_ offered an adequate prediction of mortality (ASPI AUC ROC = 0.75 (0.46–0.75); ADP AUC ROC = 0.72 (0.48–0.72); TRAP AUC ROC = 0.75 (0.54–0.75); Table 2).

To increase the predictive power of mtDNA for mortality, we analyzed multiple combinations of the mtDNA levels with the ROTEM and platelet impedance aggregometric parameters. The multiplication of the mtDNA level with the EXTEM MCF at t_24_ revealed an AUC ROC of 0.90 (0.75–0.90) for in-hospital mortality, and a cut off value of 33,617 was associated with a sensitivity of 0.86 and a specificity of 1.00 (Figure 4). Furthermore, the combination of the mtDNA and TRAP levels at t_24_ showed an AUC ROC of 0.88 (0.72–0.88) (Figure 4). A sensitivity of 0.86 and a specificity of 1.00 were calculated for a cut-off value of 43,012. The results of all analyses are shown in Table 2.

**Figure 5:**
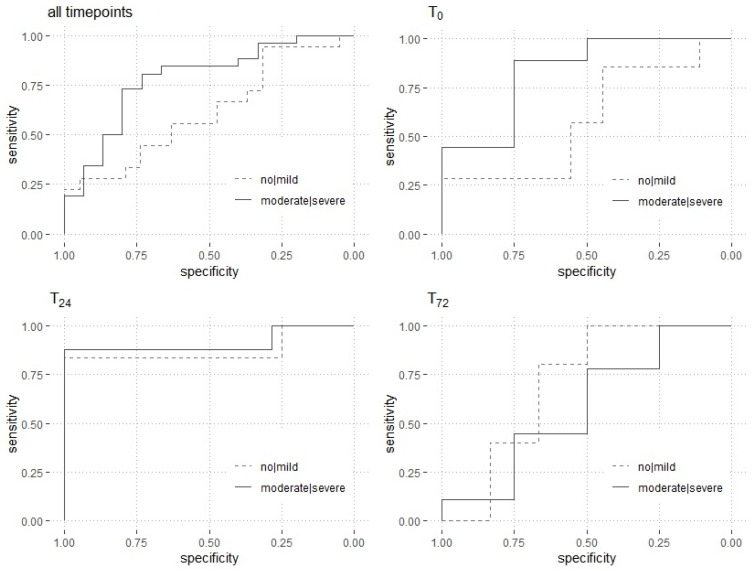
Prediction of in-hospital mortality in ARDS subgroups. The receiver operating characteristic curves are shown for the mtDNA levels, divided into ARDS-groups (no/mild and moderate/severe). The curves are presented for each time point (t_0_, t_24_ and t_72_) individually, as well as for all timepoints. Abbreviations: ARDS = acute respiratory distress syndrome.

Additionally, the COVID-19 patients were divided into adult respiratory distress syndrome (ARDS) subgroups (no/mild and moderate/severe) according to the Berlin definition of ARDS, and the prediction of hospital mortality was calculated [29]. Except for t_72_, ROC analysis showed a sufficient prediction of mortality in the moderate/severe ARDS subgroup. The results of the ARDS subanalysis are presented in Figure 5 and Table 3.

## 4. Discussion

Until today, little data has been available to investigate free circulating plasma mtDNA levels in critically ill patients with COVID-19 and their correlation with coagulatory function [15,30,31]. Compared with the healthy controls, patients with COVID-19 showed an elevated level of ND1 mtDNA, an impaired platelet function, a plasmatic hypercoagulability (which is reflected by an increased A10, A20 and MCF) and impaired fibrinolysis. COVID-19 is known to trigger hypercoagulability with a simultaneous decrease in fibrinolytic capacity, which can be identified using thromboelastography [15,16,17,18,19,20,21,27,28,29,30]. However, most previous studies have reported an aberrance of thromboelastographic results to standard values. In contrast, our study demonstrated that hypercoagulability and impaired fibrinolysis were also present in the patient group compared with the control group, matched by age, sex and pre-existing disorders.

Since immunothrombosis plays a pivotal role in COVID-19, biomarkers of the early innate immune and coagulation response are of interest for the development of diagnostic biomarkers. In this context, neutrophil extracellular traps (NETs) are key players, and have therefore already been investigated [32,33]. It has recently been demonstrated that the quantification of NETs, as well as their surrogate parameters (e.g., cell-free nucleic acids), can offer sufficient predictive power for identifying critically ill patients suffering from COVID-19, and moreover, that they can potentially display prognostic biomarkers for survival [34,35,36,37]. In contrast, the release of mtDNA during COVID-19 has been investigated significantly less, even though it is related to NETs and immunothrombosis. Therefore, the present study aimed to quantify mtDNA [38,39,40].

MtDNA displays damage-associated molecular patterns, leading to the activation of neutrophils and platelets via Toll-like receptor 9 (TLR 9) [41]. Accordingly, mtDNA might be of interest in the context of COVID-19-associated hypercoagulability. We observed an early elevation of plasma ND1 mtDNA levels lasting over 24 h in the patient group, compared with the control group. However, the data at the final time point of 72 h (Figure 2) failed to reach statistical significance, most possibly owing to the small number of included cases. Another possible explanation might be clinical improvements in the patients after three days of ICU treatment. Surprisingly, we were unable to identify any associations between the plasma ND1 mtDNA levels and thromboelastographic parameters. 

Scozzi et al. have comparably investigated the role of mtDNA in patients with COVID-19. They measured the plasma mtDNA levels of patients with COVID-19 at hospital admission, and reported that the levels were higher in patients requiring ICU therapy [15]. Since Scozzi et al. analyzed the levels of mtDNA encoding for cytochrome B, the absolute amounts of mtDNA cannot directly be compared with those in our study. With a lack of other comparable studies, due to different measurements of mtDNA, the amount of circulating ND1 mtDNA can be evaluated against cases of septic shock, which we have previously investigated [13]. In our previous study, no correlation between the plasma mtDNA levels and fibrinogen-dependent thromboelastographic parameters could be found in patients with septic shock [13]. Furthermore, the ND1 mtDNA levels were elevated after 72 h in patients with septic shock, which might be explained by a more severe illness (sequential organ failure assessment score at 72 h: 6.5 [4.0–8.3] vs. 9 [5.5–14.5]) or the limited number of patients included [13,42].

Why clinically relevant coagulation parameters do not correlate with the amount of mtDNA—although they are both elevated in patients with COVID-19 and closely connected from a pathophysiological point of view—remains unclear. One possible explanation might be the TLR-9 dependence of mtDNA, which triggers platelets rather than activating them. Since platelet function cannot be assessed using thromboelastography, we investigated the correlation of the mtDNA level with platelet function, as measured on impedance aggregometry. Analogous to the thromboelastographic results, no association was identified, although a significant impairment of platelet function was detected.

The data presented may be limited, but they are in line with those reported by Herrmann et al., who performed platelet impedance aggregometry in 18 patients with COVID-19 admitted to an ICU, and also found ASPI- and ADP-stimulated platelet function impairments [43]. Heinz et al. compared the aggregometric results of 27 critically ill patients with COVID-19 with those of 12 healthy controls. Interestingly, they also detected no differences in the TRAP levels; however, the ADP levels significantly decreased in patients with COVID-19. Contrary to our findings, however, Heinz et al. revealed no differences in the ASPI levels, which might be explained by the lack of matching with healthy controls in their study [44]. The small sample size of all mentioned studies could also account for the difference in the ASPI levels. To date, no known causative mechanisms could explain the preserved TRAP level in contrast to the ADP- and ASPI-stimulated platelet function impairments.

The current study also evaluated the plasma ND1 mtDNA levels as predictive biomarkers for patients with COVID-19 requiring ICU treatment. The peak median plasma levels of mtDNA, which were measured within 24 h after admission, were found to be strongly predictive of in-hospital mortality. However, the plasma mtDNA levels over 72 h, as well as at the single time points, were not predictive of mortality, indicating that the time point of the blood sample collection is important for predicting mortality based on mtDNA levels. Another explanation might involve the high data variability within the plasma mtDNA levels. Recently, mtDNA was evaluated in COVID-19 patients with ARDS [30]. An explorative study by Hepokoski et al. showed that increased plasma levels of mtDNA encoding for cytochrome B and ND1 were higher in COVID-19 patients with moderate or severe ARDS. As in our study, only a limited number of patients (*n* = 20) were enrolled, and a strong increase in mtDNA was indicated. In another study by Andargie et al., the plasma mtDNA levels of 85 patients with COVID-19 were measured [31]. This study was able to demonstrate that mtDNA levels were higher in patients suffering from COVID-19 compared to healthy controls, as well as to patients with other virus-related diseases (influenza and respiratory syncytial virus). In contrast to our study, however, the mtDNA plasma levels were not sufficiently able to predict mortality. Nevertheless, our results support the findings of both studies by demonstrating a significant increase of mtDNA in critically ill patients suffering from COVID-19, which was predictive of hospital mortality in patients with moderate or severe ARDS in the summarized data of all COVID-19 time points at t_0_ and t_24_, respectively. This is of particular interest as both prior studies used a droplet digital PCR, allowing absolute quantification of mtDNA without the need for DNA isolation or the generation of a standard curve. Moving forward, validation studies will be necessary, but our study results encourage the use of droplet digital PCR for translational studies at the ICU.

Another comparable study by Scozzi et al. showed lower predictive validity (AUC ROC = 0.68 [0.54–0.81] vs. 0.90 [0.75–0.90]) [15]. However, the time points of blood collection were not comparable. While Scozzi et al. collected blood samples after hospital admission, we collected samples after ICU admission, indicating a more severe illness at our time point of blood collection. As explained above, assessing the peak plasma mtDNA levels could be critical for achieving sufficient predictive validity. This might also explain why elevated levels of mtDNA were associated with increased mortality in patients without COVID-19 admitted to the ICU [14]. Since COVID-19 patients develop hypercoagulability and impaired platelet function as a sign of immunothrombosis, it seemed reasonable to investigate the predictive performance of mtDNA combined with EXTEM and FIBTEM MCF—the aggregometric parameters (like the ASPI, ADP and TRAP tests). Herein, the combination of the ND1 mtDNA level with the thromboelastographic and platelet aggregometric parameters did not increase the predictive power. The combinations of the FIBTEM MCF with the ND1 mtDNA levels and the TRAP levels with the ND1 mtDNA levels 24 h after ICU admission revealed an AUC ROC of 0.88, which is reduced to the predictive power of the ND1 mtDNA level alone. The ND1 mtDNA levels among all time points showed a high variance ranging from 28 to 7388 copies/µL, which might offer an explanation as to why the predictive performance of the mtDNA level could not be increased by the combination with the thromboelastographic and platelet aggregometric parameters [15].

This study has some limitations. First, due to the explorative character of this study, no sample size calculation was feasible. Nevertheless, differences in the levels of ND1 mtDNA between the patient and control groups reached statistical significance. Second, all patients admitted to the ICU with positive PCR test results for SARS-CoV-2 were included in this study, indicating that the disease severity was heterogeneous; however, not all patients demonstrated a severe form of ARDS. Nevertheless, a Murray score of 1.9 [1.3–2.5] was calculated at admission to the ICU, and 58.3% of the patients with COVID-19 received invasive ventilation after 72 h. Third, due to the explorative character of this study and in order to achieve a high grade of divergence from critically ill COVID-19 patients, the control group consisted of healthy probands, matched by age, sex and pre-existing disorders. Consequently, the next step in validating mtDNA as a diagnostic biomarker for COVID-19 must contain a comparison of critically ill patients with and without COVID-19. Lastly, because no sequencing data for SARS-CoV-2 were available in this study, the influence of possible mutations remains unclear. New studies on patients with other SARS-CoV-2 mutations are needed to confirm our findings.

## 5. Conclusions

In summary, critically ill COVID-19 patients present an early increase in the plasma levels of ND1 mtDNA lasting over 24 h. They also show platelet function and fibrinolysis impairments and hypercoagulability, but these do not correlate with the plasma levels of mtDNA. The peak plasma mtDNA levels can be used as a predictive biomarker for in-hospital mortality. However, the combination with coagulatory parameters does not improve the predictive validity.

## Figures and Tables

**Table 1:** Description of the study cohorts. Since some patients deceased or were transferred to the ward, the analysis consists of 29 (t_0_), 25 (t_24_) and 24 (t_72_) patients, respectively. Furthermore, 3 patients received no anticoagulation at t_72_.

		Patients with COVID-19 (*n* = 29)	Controls (*n* = 29)
**General characteristics**			
Age (year)		70 (59–80)	70 (58–79)
Male sex (%)		65.5	65.5
BMI (kg/m^2^)		28.4 (24.2–30.5)	29.7 (26.8–32.0)
ARDS	Admission	7 (24.1%), 7 (24.1%), 9 (31.0%), 6 (20.7%)	NA
(no, mild)	24 h	5 (20.0%), 3 (12.0%), 13 (52.0%), 4 (16.0%)	NA
(moderate, severe)	72 h	5 (20.8%), 4 (16.7%), 13 (54.1%), 2 (8.3%)	NA
Murray score	Admission	1.9 (1.3–2.5)	NA
	24 h	1.8 (1.3–2.5)	NA
	72 h	2.3 (1.7–2.6)	NA
SOFA score	Admission	7.0 (5.0–9.0)	NA
	24 h	6.0 (5.0–8.0)	NA
	72 h	6.5 (4.0–8.3)	NA
In-hospital mortality		16 (55.2%)	0 (0.0%)
**Pre-existing diseases**			
CAD		8 (27.6%)	8 (27.6%)
Arterial hypertension		25 (86.2%)	25 (86.2%)
Diabetes mellitus		14 (48.3%)	14 (48.3%)
Chronic kidney disease		5 (17.2%)	5 (17.2%)
**Anticoagulation**			
Prophylactic	Admission	16 (55.2%)	0 (0.0%)
	24 h	9 (36.0%)	NA
	72 h	8 (33.3%)	NA
Therapeutic	Admission	13 (44.8%)	0 (0.0%)
	24 h	16 (64.0%)	NA
	72 h	13 (54.2%)	NA
Heparin	Admission	5.7 (5.0–10.0)	0 (0–0)
(I.U./kg/d)	24 h	7.3 (4.8–11.4)	NA
	72 h	8.7 (4.3–12.3)	NA
Enoxaparin	Admission	1.1 (0.8–1.5)	0 (0–0)
(mg/kg/d)	24 h	1.4 (1.0–1.8)	NA
	72 h	1.4 (1.1–1.9)	NA
**ICU treatment**			
NIV	Admission	11 (37.9%)	
	24 h	11 (44.0%)	
	72 h	5 (20.8%)	
INV	Admission	7 (24.1%)	
	24 h	9 (36.0%)	
	72 h	14 (58.3%)	
ECMO	Admission	1 (3.4%)	
	24 h	2 (8.0%)	
	72 h	3 (12.5%)	
Dialysis	Admission	3 (10.3%)	
	24 h	5 (20.0%)	
	72 h	7 (29.2%)	

Abbreviations: BMI = body mass index; CAD = coronary artery disease; ECMO = extracorporeal membrane oxygenation; I.U. = International units. NA = not applicable; NIV = non-invasive ventilation; INV = invasive ventilation; SOFA = sequential organ failure assessment.

**Table 2:** AUC with 95% confidence interval for the prediction of mortality.

Parameter	Timepoint	AUC	Cut off	Specificity	Sensitivity
mtDNA level	All	0.73 (0.61–0.73)	638	0.88	0.48
mtDNA level	t_0_	0.73 (0.54–0.73)	681	0.92	0.50
mtDNA level	t_24_	0.90 (0.75–0.90)	420	1.00	0.86
mtDNA level	t_72_	0.50 (0.25–0.50)	467	0.60	0.57
FIBTEM MCF	All	0.63 (0.50–0.63)	42	0.44	0.79
FIBTEM MCF	t_0_	0.65 (0.43–0.65)	32	0.73	0.57
FIBTEM MCF	t_24_	0.58 (0.35–0.58)	21	1.00	0.21
FIBTEM MCF	t_72_	0.66 (0.42–0.66)	38	0.70	0.64
EXTEM MCF	All	0.66 (0.54–0.66)	71	0.91	0.45
EXTEM MCF	t_0_	0.66 (0.43–0.66)	71	0.91	0.50
EXTEM MCF	t_24_	0.67 (0.46–0.67)	71	0.91	0.50
EXTEM MCF	t_72_	0.65 (0.42–0.65)	79	0.40	0.86
ASPI	All	0.62 (0.43–0.62)	49	0.88	0.50
ASPI	t_0_	0.51 (0.15–0.51)	48	0.80	0.43
ASPI	t_24_	0.57 (0.22–0.57)	38	0.83	0.57
ASPI	t_72_	0.75 (0.46–0.75)	53	1.00	0.50
ADP	All	0.58 (0.44–0.58)	34	0.94	0.28
ADP	t_0_	0.55 (0.29–0.55)	108	0.91	0.36
ADP	t_24_	0.38 (0.12–0.38)	79	0.55	0.55
ADP	t_72_	0.72 (0.48–0.72)	51	0.89	0.60
TRAP	All	0.58 (0.44–0.58)	73	0.84	0.37
TRAP	t_0_	0.50 (0.26–0.50)	81	0.73	0.43
TRAP	t_24_	0.46 (0.22–0.46)	60	0.18	0.93
TRAP	t_72_	0.75 (0.54–0.75)	74	1.00	0.54
mtDNA × FIBTEM MCF	All	0.69 (0.57–0.69)	9617	0.66	0.71
mtDNA × FIBTEM MCF	t_0_	0.69 (0.47–0.69)	8477	0.64	0.79
mtDNA × FIBTEM MCF	t_24_	0.88 (0.73–0.88)	17,869	1.00	0.79
mtDNA × FIBTEM MCF	t_72_	0.55 (0.30–0.55)	9186	0.80	0.43
mtDNA × EXTEM MCF	All	0.72 (0.60–0.72)	21,970	0.69	0.69
mtDNA × EXTEM MCF	t_0_	0.71 (0.50–0.71)	42,896	0.82	0.57
mtDNA × EXTEM MCF	t_24_	0.90 (0.75–0.90)	33,617	1.00	0.86
mtDNA × EXTEM MCF	t_72_	0.51 (0.26–0.51)	35,936	0.60	0.57
mtDNA × ASPI	All	0.57 (0.38–0.57)	47,136	0.81	0.45
mtDNA × ASPI	t_0_	0.60 (0.25–0.60)	56,470	0.80	0.57
mtDNA × ASPI	t_24_	0.71 (0.40–0.71)	45,868	1.00	0.57
mtDNA × ASPI	t_72_	0.68 (0.36–0.68)	14,943	1.00	0.38
mtDNA × ADP	All	0.68 (0.55–0.68)	32,492	0.81	0.63
mtDNA × ADP	t_0_	0.73 (0.49–0.73)	34,491	0.82	0.73
mtDNA × ADP	t_24_	0.83 (0.63–0.83)	33,900	1.00	0.73
mtDNA × ADP	t_72_	0.60 (0.32–0.60)	22,243	0.78	0.60
mtDNA × TRAP	All	0.70 (0.57–0.70)	48,917	0.81	0.61
mtDNA × TRAP	t_0_	0.72 (0.51–0.72)	48,989	0.91	0.57
mtDNA × TRAP	t_24_	0.88 (0.72–0.88)	43,012	1.00	0.86
mtDNA × TRAP	t_72_	0.60 (0.35–0.60)	30,562	0.78	0.54

Abbreviations: ADP = adenosine diphosphate; AUC = area under the curve; ASPI = arachidonic acid; EXTEM = extrinsically activated thromboelastometry; FIBTEM = fibrinogen-based thromboelastometry; MCF = maximum clot firmness; mtDNA = mitochondrial DNA; TRAP = thrombin receptor-activating peptide.

**Table 3:** AUC with 95% confidence interval for the prediction of in-hospital mortality in ARDS subgroups.

Parameter	Timepoint	ARDS-Group	AUC	Cut Off	Specificity	Sensitivity
mtDNA level	All	no/mild	0.62 (0.43–0.62)	70	0.32	0.94
mtDNA level	t_0_	no/mild	0.59 (0.28–0.59)	68	0.44	0.86
mtDNA level	t_24_	no/mild	0.88 (0.62–0.88)	369	1.00	0.83
mtDNA level	t_72_	no/mild	0.70 (0.35–0.70)	533	0.50	1.00
mtDNA level	All	severe/moderate	0.78 (0.63–0.78)	282	0.73	0.81
mtDNA level	t_0_	severe/moderate	0.83 (0.55–0.83)	275	0.75	0.89
mtDNA level	t_24_	severe/moderate	0.91 (0.73–0.91)	420	1.00	0.88
mtDNA level	t_72_	severe/moderate	0.58 (0.17–0.58)	224	0.50	0.78

Abbreviations: ARDS = acute respiratory distress syndrome; AUC = area under the curve; mtDNA = mitochondrial DNA.

## Data Availability

Not applicable.

## Supplementary Materials

The following supporting information can be downloaded at: https://www.mdpi.com/article/10.3390/jcm11237161/s1, Table S1, Overview on the laboratory parameters; Table S2, Description of the study cohorts; Table S3, Results of ROTEM Analysis; Figure S1: Time course of thromboelastometry A10; and Figure S2: Time course of thromboelastometry A20.
