# Supplementary material for: Peak Plasma Levels of mtDNA Serve as a Predictive Biomarker for COVID-19 in-Hospital Mortality
Source: J Clin Med. 2022 Dec 1;11(23):7161. doi: 10.3390/jcm11237161 (PMC9740249; doi:10.3390/jcm11237161)
Supplement: Supplementary file 1 [file jcm-11-07161-s001.zip › jcm-1998469-supplementary.pdf]

Table S1: Overview on the laboratory parameters. All data are shown as median with interquartile range or percentage. *Abbreviations: CRP = C reactive protein; GFR = glomerular filtration rate; GOT = glutamate oxalate transaminase; GPT = glutamate pyruvate transaminase; INR = international normalized ratio; LDH = lactate dehydrogenase; PCT = procalcitonin; U = Units.*

		COVID-19 (n = 29)	Controls (n = 29)	*p*-Value
Leucocytes (10^9/L)	Admission	10.0 [7.2 – 15.7]	7.6 [6.6 – 9.1]	0.73
	24 h	9.6 [7.5 – 16.5]	NA	0.38
	72 h	12.9 [9.3 – 18.9]	NA	0.009
Lymphocytes (10^9/L)	Admission	0.46 [0.31 – 0.78]	NA	
	24 h	0.40 [0.30 – 0.53]	NA	
	72 h	0.63 [0.34 – 0.96]	NA	
Thrombocytes (10^9/L)	Admission	210 [151 – 286]	250 [212 – 281]	1.00
	24 h	215 [171 – 265]	NA	1.00
	72 h	245 [151 – 332]	NA	1.00
Neutrophilic granulocytes (10^9/L)	Admission	9.4 [6.2 – 15.1]	NA	
	24 h	8.3 [7.4 – 11.0]	NA	
	72 h	12.1 [8.4 – 17.8]	NA	
CRP (mg/L)	Admission	155.4 [131.1 – 205.3]	2.1 [0.5 – 5.3]	< 0.001
	24 h	149.8 [121.1 – 231.4]	NA	< 0.001
	72 h	131.7 [67.8 – 125.4]	NA	< 0.001
PCT (µg/L)	Admission	4.7 [2.6 – 7.3]	NA	
	24 h	5.7 [3.8 – 11.6]	NA	
	72 h	5.9 [4.3 – 10.2]	NA	
Interleukin 6 (pg/mL)	Admission	49.5 [35.1 – 149.8]	NA	
	24 h	17.1 [13.6 – 37.4]	NA	
	72 h	20.9 [0.0 – 54.8]	NA	
Ferritin (ng/mL)	Admission	1171 [432 – 1905]	240 [127 – 887]	1.00
	24 h	1516 [427 – 2487]	NA	1.00
	72 h	1159 [451 – 2163]	NA	1.00
INR	Admission	1.0 [1.0 – 1.1]	0.9 [0.9 – 0.9]	0.55
	24 h	1.0 [1.0 – 1.2]	NA	0.25
	72 h	1.0 [1.0 – 1.1]	NA	0.93
Fibrinogen (g/L)	Admission	4.8 [4.1 – 6.2]	NA	
	24 h	5.4 [4.4 – 6.7]	NA	
	72 h	4.9 [3.9 – 6.0]	NA	
D-Dimers (µg/mL)	Admission	1.8 [0.8 – 4.8]	NA	
	24 h	1.7 [0.9 – 3.5]	NA	
	72 h	2.5 [0.8 – 5.3]	NA	
Creatinine (mg/dL)	Admission	1.2 [0.9 – 2.2]	0.9 [0.8 – 1.0]	1.00
	24 h	1.2 [0.9 – 2.0]	NA	1.00
	72 h	1.6 [1.0 – 2.5]	NA	1.00
Urea (mg/dL)	Admission	68 [49 – 82]	31 [26 – 90]	1.00
	24 h	69 [55 – 98]	NA	1.00
	72 h	103 [66 – 138]	NA	0.07
GFR (mL/min)	Admission	49 [31 – 90]	86 [57 – 105]	1.00
	24 h	52 [30 – 90]	NA	1.00

	72 h	41 [24 – 79]	NA	0.41
GOT (U/L)	Admission	64 [37 – 105]	25 [19 – 30]	0.92
	24 h	73 [36 – 128]	NA	1.00
	72 h	64 [39 – 118]	NA	0.19
GPT (U/L)	Admission	35 [27 – 57]	23 [17 – 36]	0.46
	24 h	41 [31 – 69]	NA	0.56
	72 h	45 [33 – 76]	NA	0.12
LDH (U/L)	Admission	508 [315 – 769]	210 [208 – 253]	0.34
	24 h	400 [288 – 631]	NA	1.00
	72 h	487 [348 – 593]	NA	0.37

Table S2. Description of the study cohorts. *Abbreviations: BMI = body mass index; CAD = coronary artery disease; ECMO = extracorporeal membrane oxygenation; I.U. = International units; NA = not applicable; NIV = non-invasive ventilation; INV = invasive ventilation; SOFA = sequential organ failure assessment.*

		Deceased Patients (n = 16)
**General characteristics**		
Age (year)		73 [67–82]
Male sex (%)		56.3
BMI (kg/m^2)		28.4 [24.3–31.1]
ARDS (no, mild, moderate, severe)	Admission	5 (31.3%), 2 (12.5%), 5 (31.3%), 4 (25.0%)
	24 h	4 (28.6%), 2 (14.3%), 6 (42.9%), 2 (14.3%)
	72 h	2 (14.3%), 3 (21.4%), 7 (50.0%), 2 (14.3%)
Murray score	Admission	2.0 [1.3–2.8]
	24 h	1.8 [1.5–2.6]
	72 h	2.3 [2.0–2.9]
SOFA score	Admission	7.0 [5.3–9.8]
	24 h	5.5 [4.8–9.5]
	72 h	7.5 [4.0–9.3]
**Pre-existing diseases**		
CAD		6 (37.5%)
Arterial hypertension		14 (87.5%)
Diabetes mellitus		8 (50.0%)
Chronic kidney disease		3 (18.8%)
**Anticoagulation**		
Prophylactic	Admission	12 (75.0%)
	24 h	7 (50.0%)
	72 h	6 (50.0%)
Therapeutic	Admission	4 (25.0%)
	24 h	7 (50.0%)
	72 h	6 (50.0%)
Heparin (I.U./kg/d)	Admission	5.0 [4.4–10.0]
	24 h	6.8 [4.5–11.0]
	72 h	8.7 [3.2–14.9]
Enoxaparin (mg/kg/d)	Admission	0.8 [0.6–1.2]
	24 h	1.0 [0.9–1.7]

	72 h	1.1 [0.7–1.5]
**ICU treatment**		
NIV	Admission	7 (43.8%)
	24 h	7 (50.0%)
	72 h	4 (28.6%)
INV	Admission	5 (31.3%)
	24 h	5 (35.7%)
	72 h	10 (71.4%)
ECMO	Admission	0 (0.0%)
	24 h	0 (0.0%)
	72 h	1 (7.1%)
Dialysis	Admission	1 (6.3%)
	24 h	2 (14.3%)
	72 h	4 (28.6%)

Table S3: Results of ROTEM Analysis. *Abbreviations: APTEM = aprotinin-based thromboelastometry; CFT = clot formation time; CT = clotting time; EXTEM = extrinsically activated thromboelastometry; FIBTEM = fibrinogen-based thromboelastometry; INTEM = intrinsically activated thromboelastometry; MCF = maximum clot firmness; ML = maximum lysis.*

		COVID-19 (n = 29)	Controls (n = 29)
EXTEM CT (sec)	Admission	87 [72 – 125]	72 [66 – 84]
	24 h	87 [77 – 94]	
	72 h	87 [76 – 99]	
EXTEM CFT (sec)	Admission	59 [44 – 73]	74 [53 – 95]
	24 h	55 [41 – 65]	
	72 h	51 [45 – 72]	
EXTEM α (°)	Admission	78 [75 – 81]	75 [71 – 79]
	24 h	79 [77 – 82]	
	72 h	80 [76 – 81]	
EXTEM A10 (mm)	Admission	69 [62 – 73]	61 [53 – 69]
	24 h	71 [64 – 73]	
	72 h	70 [65 – 75]	
EXTEM A20 (mm)	Admission	74 [67 – 77]	67 [60 – 74]
	24 h	75 [69 – 77]	
	72 h	74 [72 – 79]	
EXTEM MCF (mm)	Admission	75 [69 – 77]	68 [61 – 75]
	24 h	76 [69 – 77]	
	72 h	75 [72 – 79]	
EXTEM ML (%)	Admission	0 [0 – 3]	10 [3 – 11]
	24 h	0 [0 – 2]	
	72 h	0 [0 – 1]	
INTEM CT (sec)	Admission	231 [208 – 281]	216 [183 – 244]
	24 h	249 [196 – 299]	
	72 h	254 [200 – 324]	
INTEM CFT (sec)	Admission	52 [44 – 74]	69 [58 – 83]

	24 h	53 [44 – 71]	
	72 h	53 [45 – 71]	
INTEM α (°)	Admission	80 [75 – 81]	76 [73 – 78]
	24 h	79 [76 – 81]	
	72 h	79 [77 – 81]	
INTEM A10 (mm)	Admission	69 [61 – 70]	57 [55 – 65]
	24 h	68 [62 – 71]	
	72 h	70 [63 – 71]	
INTEM A20 (mm)	Admission	73 [68 – 74]	62 [60-69]
	24 h	72 [67 – 75]	
	72 h	73 [68 – 76]	
INTEM MCF (mm)	Admission	74 [67 – 75]	64 [61 – 70]
	24 h	73 [68 – 75]	
	72 h	74 [68 – 76]	
INTEM ML (%)	Admission	0 [0 – 3]	8 [4 – 13]
	24 h	0 [0 – 1]	
	72 h	0 [0 – 1]	
FIBTEM CT (sec)	Admission	80 [71 – 114]	69 [64 – 75]
	24 h	80 [69 – 93]	
	72 h	79 [71 – 97]	
FIBTEM CFT (sec)	Admission	90 [60 – 124]	312 [115 – 760]
	24 h	99 [60 – 124]	
	72 h	69 [57 – 93]	
FIBTEM α (°)	Admission	75 [73 – 79]	75 [71 – 78]
	24 h	77 [73 – 81]	
	72 h	78 [73 – 80]	
FIBTEM A10 (mm)	Admission	31 [25 – 39]	20 [17 – 27]
	24 h	33 [25 – 39]	
	72 h	33 [29 – 38]	
FIBTEM A20 (mm)	Admission	35 [28 – 42]	22 [18 – 28]
	24 h	35 [27 – 42]	
	72 h	36 [31 – 40]	
FIBTEM MCF (mm)	Admission	35 [28 – 43]	23 [18 – 28]
	24 h	35 [27 – 43]	
	72 h	38 [33 – 41]	
FIBTEM ML (%)	Admission	0 [0 – 0]	4 [0 – 8]
	24 h	0 [0 – 0]	
	72 h	0 [0 – 0]	
APTEM CT (%EXTEM CT)	Admission	97 [85 – 101]	93 [86 – 100]
	24 h	92 [88 – 101]	
	72 h	96 [93 – 99]	
APTEM CFT (%EXTEM CFT)	Admission	107 [96 – 123]	96 [91 – 108]
	24 h	106 [95 – 113]	

	72 h	102 [93 – 108]	
APTEM α (%EXTEM α)	Admission	99 [98 – 100]	100 [100 – 103]
	24 h	100 [99 – 101]	
	72 h	100 [99 – 102]	
APTEM A10 (%EXTEM A10)	Admission	97 [96 – 100]	100 [97 – 101]
	24 h	98 [96 – 100]	
	72 h	100 [97 – 101]	
APTEM A20 (%EXTEM A20)	Admission	97 [97 – 99]	100 [98 – 100]
	24 h	99 [97 – 100]	
	72 h	99 [97 – 100]	
APTEM MCF (%EXTEM MCF)	Admission	97 [97 – 99]	100 [98 – 101]
	24 h	99 [97 – 100]	
	72 h	99 [97 – 100]	
APTEM ML (%EXTEM ML)	Admission	100 [100 – 100]	100 [82 – 100]
	24 h	100 [100 – 100]	
	72 h	100 [96 – 100]	

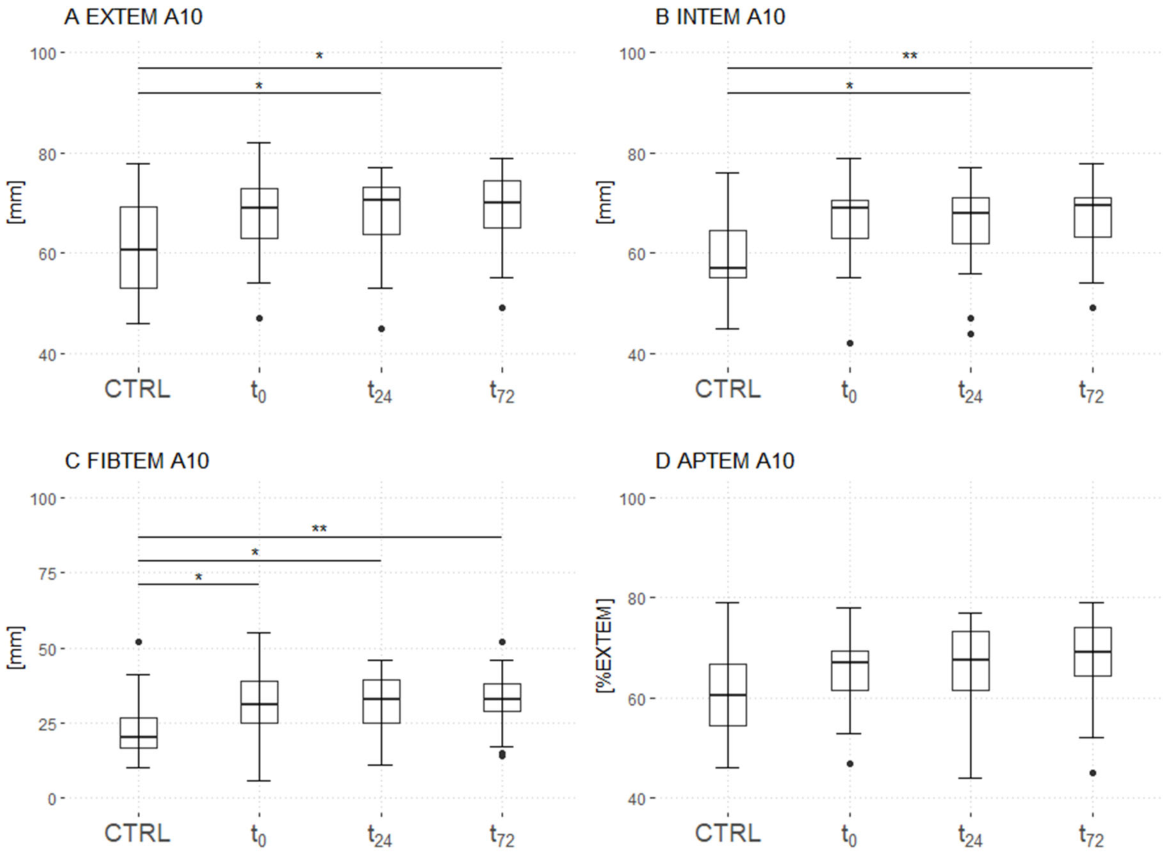

Figure S1: Time course of thromboelastometry A10. Patients with COVID-19 presented a significantly increased A10 24 and 72 hours after admission to the ICU in the EXTEM (A) and INTEM assays (B) compared with controls. In the FIBTEM assay (C), the A10 increased in patients with COVID-19 at all timepoints compared with that in controls. In the APTEM assay (D), no differences of A10 were recorded compared with the control group. Asterisks display the degree of statistical significance: *: $p < 0.05$, **: $p < 0.01$. *Abbreviations: A10 = amplitude 10*

minutes after clotting time; APTEM = aprotinin-based thromboelastometry; CTRL = control group; EXTEM = extrinsically activated thromboelastometry; FIBTEM = fibrinogen-based thromboelastometry; INTEM = intrinsically activated thromboelastometry.

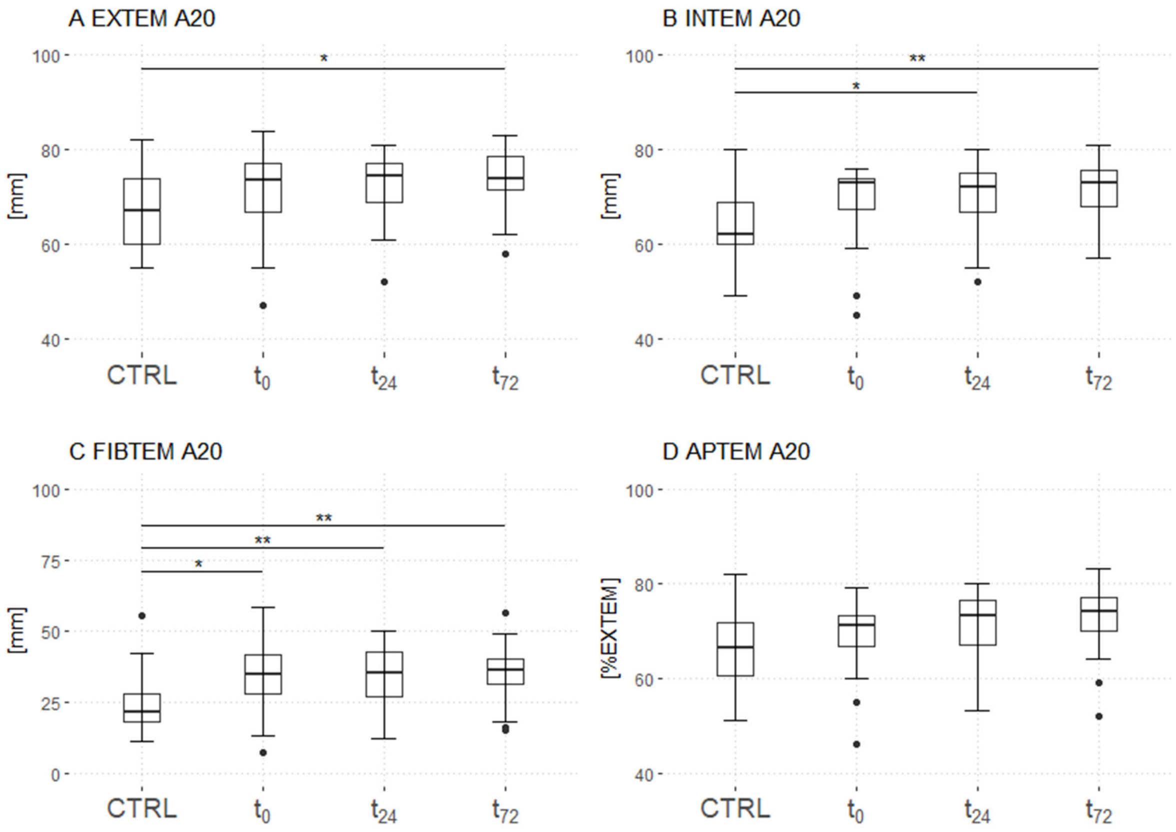

Figure S2: Time course of thromboelastometry A20. Patients with COVID-19 presented a significantly increased A20 72 hours after admission to the ICU in the EXTEM (A) and INTEM assays (B) compared with controls. Further, elevated values of A20 were also measured at t_{24} in INTEM assay (B) compared with control group. In the FIBTEM assay (C), the A20 increased in patients with COVID-19 at all timepoints compared with that in controls. In the APTEM assay (D), no differences of A20 were recorded compared with the control group. Asterisks display the degree of statistical significance: *: $p < 0.05$, **: $p < 0.01$. *Abbreviations: A20 = amplitude 20 minutes after clotting time; APTEM = aprotinin-based thromboelastometry; CTRL = control group; EXTEM = extrinsically activated thromboelastometry; FIBTEM = fibrinogen-based thromboelastometry; INTEM = intrinsically activated thromboelastometry.*
